# MicroRNA-106b is involved in transforming growth factor β1–induced cell migration by targeting disabled homolog 2 in cervical carcinoma

**DOI:** 10.1186/s13046-016-0290-6

**Published:** 2016-01-15

**Authors:** Yuan Cheng, Yanli Guo, Youyi Zhang, Ke You, Zijian Li, Li Geng

**Affiliations:** Department of Gynecology and Obstetrics, Peking University Third Hospital, Beijing, 100191 China; Institute of Vascular Medicine, Peking University Third Hospital, Beijing Key Laboratory of Cardiovasicular Receptors Research, Key Laboratory of Cardiovascular Molecular Biology and Regulatory Peptides, Ministry of Health and Key Laboratory of Molecular Cardiovascular Sciences, Ministry of Education, Beijing, 100191 China

**Keywords:** MicroRNA-106b, Cervical cancer, Transforming growth factor β1, Disabled homolog 2

## Abstract

**Background:**

MicroRNA-106b (miR-106b) was recently identified as an oncogene participating in cancer progression. Transforming growth factor β1(TGF-β1) is an indispensable cytokine regulating the local microenvironment, thereby promoting cervical cancer progression. However, the roles of miR-106b in cervical carcinoma progression and TGF-β1-involvement in the tumorigenesis of cervical cancer remain unknown.

**Methods:**

The expression of miR-106b in human cervical specimens was detected by real-time PCR analysis and *in situ* hybridization assay. The effect of miR-106b on cell migration was analyzed by scratch and transwell assays. TGF-β1 was used to induce cell migration. The expression of the miR-106b target gene DAB2 in human cervical tissues and cell lines were measured by qRT-PCR, western blot and immunohistochemistry. Dual-luciferase reporter assay was used to identify DAB2 as a miR-106b-directed target gene.

**Results:**

miR-106b was frequently up-regulated in human cervical carcinoma specimens and cervical cancer cell lines. Over-expression of miR-106b significantly promoted HeLa and SiHa cells migration. Likewise, inhibition of miR-106b decreased HeLa and SiHa cells migration. The multifunctional cytokine TGF-β facilitates metastasis in cervical carcinoma. miR-106b inhibitor treatment decreased the TGF-β1-stimulated migration of HeLa and SiHa cells. DAB2, a predicted target gene of miR-106b, was inhibited by TGF-β1 partly through miR-106b and was involved in TGF-β1-induced cervical cancer cell migration. The expression of DAB2 was low in cervical cancer tissues, and negatively correlated with miR-106b expression. Finally, DAB2 was identified as a miR-106b-directed target gene by dual-luciferase reporter assay.

**Conclusion:**

Our data suggest that the TGF-β1/miR-106b/DAB2 axis may be involved in the pathogenesis of cervical carcinoma.

## Background

Cervical cancer is the third most common malignant tumor in women worldwide, with recently increasing incidence among young women [1–3]. Parametrical infiltration and distant metastasis of refractory cervical carcinoma critically affects the therapeutic effect and prognosis, despite screening and surgical treatment widely used in the early stages [4, 5]. Elucidating the molecular mechanism for the invasion and metastasis of cervical cancer is urgently needed. Recently, microRNAs (miRNAs) participating in cancer progression have been a focus.

miRNAs are single-stranded RNAs of about 21–23 nt that play roles in posttranscriptional regulation of target genes in cancer development, cell proliferation, apoptosis and differentiation [6–9]. Our previous study of miRNA and mRNA microarray first revealed miR-106b as a key node in the signal transduction pathway of cervical cancer [10]. However, the biological functions of miR-106b in cervical cancer have not been well investigated.

miR-106b is a member of the miR-106b ~ 25 cluster, which is homologous to the miR-17 ~ 92 cluster known as an oncogenic miRNA family. A number of reports have clarified that miR-106b is involved in the regulation of invasion and migration in various human cancers. Yang et al. showed that miR-106b is highly expressed in gastric cancer, and its expression is negatively correlated with the survival of patients; miR-106b significantly reinforced the invasion and migration ability of gastric cancer cells [11]. The high expression of miR-106b was also associated with lymph node metastasis in breast cancer. miR-106b promotes the activation of Wnt signal and induces β-catenin to enter the nucleus, thereby enhancing the ability of tumor cell invasion and migration [12]. miR-106b has been shown to participate in activation of the TGF-β/Smad signaling pathway by inhibiting Smad7 protein expression to intensify the epithelial mesenchymal transition (EMT) in breast cancer cells [13].

Extensive studies showed that TGF-β1 produced by cervical cancer cells, is a key regulator in the local microenvironment, thereby promoting invasion and migration in cervical carcinoma [14, 15]. Here, we investigated whether miR-106b can affect the migration of cervical cancer cells induced by TGF-β1 and the mechanism. We found that miR-106b had high expression in cervical cancer and promoted the migration of cervical cancer cells. miR-106b was involved in TGF-β1-induced cell migration by targeting DAB2 in cervical carcinoma. The study further revealed the molecular mechanism of cervical cancer cell migration.

## Methods

### Tumor specimens

We collected 19 cervical cancer and 19 normal tissue samples from the Department of Obstetrics and Gynecology, Peking University Third Hospital. The tumor tissues were derived from patients with a diagnosis of cervical cancer according to the FIGO criteria. The normal tissue samples were from patients with benign gynecological diseases such as uterine fibroids and chronic cervicitis. All patients voluntarily gave their signed informed consent before the operation. This study protocol was approved by the ethics committee of Peking University Third Hospital. Fresh tissues were stored in liquid nitrogen before RNA extraction. Total RNA was isolated by using Trizol reagent. The isolated RNA was dissolved in RNase-free water and stored at −80 °C.

### Cell culture, treatment and reagents

The human cervical cancer cell lines C33A, HeLa, SiHa and Caski, which are kept in our lab, were cultured in DMEM with 10 % fetal bovine serum and 100 mg/ml penicillin/streptomycin at 37 °C in 5 % CO_2_ atmosphere. Cells were treated with TGF-β1(PeproTech, Rocky Hill, USA) at 5 ng/ml for the indicated times. Cell transfection involved use of Lipofectamine2000 (Invitrogen, Carlsbad, CA, USA) with miRNA mimics and inhibitors, pmir-GLO plasmid(Life Technologies) or DAB2 siRNA. The primer sequences were for miR-106b, 5′-AAGTGCTGACAGTG CAGATAA-3′, mature miR-106b mimic, 5′-CAAAGUGC UCAUAGUGCAGGUAG -3′ and miR-106b inhibitor, 5′-GUUUCACGAGUAUCACGUCCAUC-3′(Ribobio, Guangzhou, China). The primer sequence for DAB2 siRNA was sense, 5′-AACAAA GGAUCUGGGUCAATT-3′and antisense, 5′-UUGACCCAGAUCCUUUGU UTT-3′ (GenePharma, Shanghai). DAB2 and GAPDH antibody were purchased from Santa Cruz Biotechnology (Santa Cruz, CA).

### Real-time PCR

Total RNA was extracted by the conventional Trizol method. Reverse transcription of RNA was performed using miRcute miRNA cDNA (KR201)(TIANGEN, Beijing). Mature miR-106b was detected by using a miRNA miRcute fluorescent quantitative detection kit (FP401) (TIANGEN, Beijing).

### Western blot assay

Total protein was extracted and quantified by using the BCA detection kit. Cells were lysed in 5 × loading buffer, boiled for 5 min and denatured. Cell lysates (20 μg) were separated by 10 % SDS-PAGE and transferred to nitrocellulose membranes, which were blocked with 5 % nonfat milk and incubated with primary antibodies against DAB2(1:1000, rabbit anti-human polyclonalantibody, Santa Cruz Biotechnology, Santa Cruz, CA, USA) at 4 °C overnight. The goat anti-rabbit secondary antibody (ZSGB-BIO, Beijing) was incubated for 1 h at room temperature. The membranes were exposed by use of luminescent liquid (Millipore, Massachusetts, USA) in the darkroom, dried naturally. The densitometry of protein bands was quantified by use of Image J (NIH, Bethesda, MD, USA).

### Immunohistochemistry

The paraffin sections of tissues were dewaxed as routine. Endogenous peroxidase of tissues was removed by 3 % H_2_O_2_ for 10 min. The tissues were blocked for 30 min by goat serum working fluid (ZSGB-BIO, Beijing) and stained with anti-DAB2 antibody (1:200, Santa Cruz Biotechnology, Santa Cruz, CA) at 4 °C overnight, then goat anti-rabbitsecondary antibody (ZSGB-BIO, Beijing) for 1 h at room temperature. The tissues were stained with DAB color solution for an appropriate time (ZSGB-BIO, Beijing), dehydrated, fixed and photographed. Next, we conducted a semi-quantitative analysis by using Image-Pro Plus 6.0. First, five randomly selected fields (400×) of each stained section were photographed. The integrated optical density (IOD) and mean density (IOD SUM/area) value for positive areas of each photo were calculated. The mean density of each sample was the average of the mean density from five images. We further compared the mean density of 19 cervical cancer biopsy sections and 19 normal cervical tissues samples by *t*-test analysis.

### *In situ* hybridization

*In situ* hybridization was performed by use of the miR-106b *in situ* hybridization labeling Kit. Paraffin sections of cervical cancer tissue were deparaffinized. Endogenous enzymes are inactivated by 3%H_2_O_2_. The exposure of mRNA nucleic acid fragment involved use of 3 % citric acid diluted fresh pepsin incubated for 15 min. Then, 1 % formaldehyde fixed for 10 min. After prehybridization for 4 h in a thermostat box at 38 °C, tissue was incubated with 5′digoxigenin-labeled oligonucleotide probe detecting miR-106b and hybridized overnight. Then, tissues were washed several times and blocked for 30 min, then, incubated with anti-digoxigenin antibody for 60 min at 37 °C. After rinsing, sections were incubated with SABC and Biotin peroxidase, then, stained with DAB color solution for the appropriate time and counterstained with nuclear. The relative level of miR-106b in cervical tissues was measured by Image-Pro Plus 6.0 as for the detailed content in immunohistochemistry.

### Cell scratch and migration assays

In cell scratch assays, a 20-μl pipette tip was used to make three parallel wounds and one vertical wound in each well of 6-well plates with cells incubated at 1 × 10^6^. Cells were cultured in serum-free mediumand photographed by inverted microscopy at 0 and 24 h. In the transwell assay, cells were seeded in the upper chamber of Costar transwell culture plates (24-well plates, 8 μm) and cultured in serum-free medium. DMEM with 20 % fetal bovine serum was added to the lower chamber. After 24 h, the chamber was washed 3 times with 1 × PBS. The membrane in the lower chamber was fixed in 4 % paraformaldehyde and stained with crystal violet. The cells on the upper membrane that did not migrate were wiped with a cotton swab. The number of migrated cells was counted under a microscope in at least five fields.

### Dual-luciferase reporter assay

The DAB2 gene 3′UTR with 166 bp sequences including predicted miR-106b binding sites was amplified by PCR from human genomic DNA. The amplified sequences were inserted into cloning sites of the pmir-GLO vector at SacI and SalI sites and verified by sequencing. The mutant DAB2 3′UTR vector was constructed in the dual luciferase reporter gene pmir-GLO vector with miR-106b matching nucleotides “GCACTTT” replaced with “TATAGGG” (Life Technologies, Shanghai). HEK-293A cells in 24-well plates were transfected with the wild-type and mutant DAB2 luciferase reporter vector and miR-106b mimics. Luciferase assays involved use of the Luciferase Reporter Gene Assay Kit (Promega) and activities were normalized to Renilla luciferase activity.

#### Statistical analysis

Data are expressed as mean ± SEM and were analyzed by use of GraphPad Prism 5. Two groups were compared by Student’s t-test, and multiple groups by One Way ANOVA, with further two group comparison by Multiple Comparison Test Tukey’s test. When the variance of the two groups was not homogeneous, non parametric tests were used. Correlations were examined by Spearman correlation analysis. *P* < 0.05 was considered statistically significant.

## Results

### 1. miR-106b was up-regulated in human cervical carcinoma

**Fig. 1:**
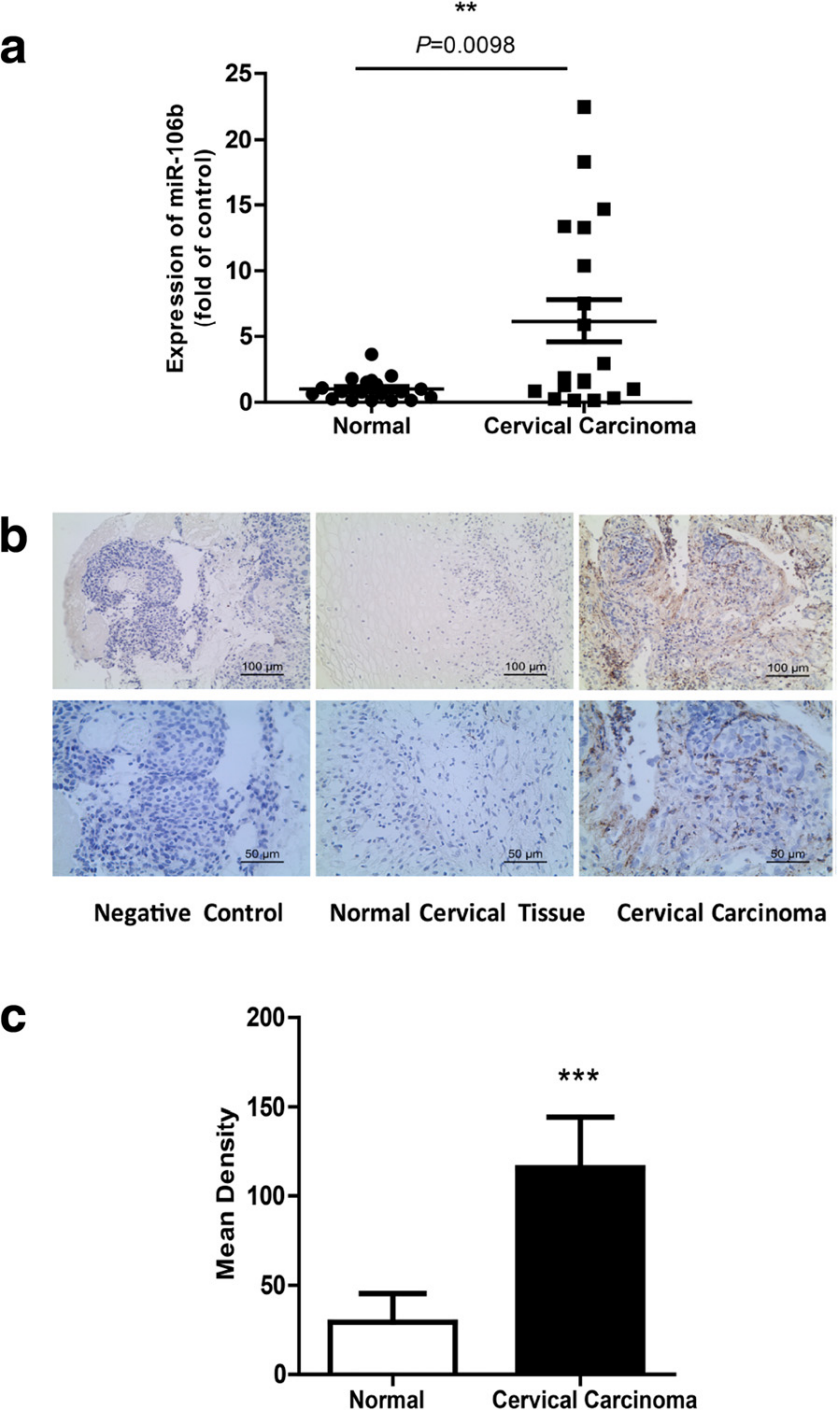
High expression of miR-106b in human cervical tissues detected by Q-PCR and *in situ* hybridization. **a** Real-time PCR analysis of the mRNA expression of miR-106b in 19 human cervical carcinoma tissues and 19 normal cervical samples. U6 small nuclear RNA was an internal control. Each point represents 1 sample. The horizontal bar is the mean and whiskers are SEM. ^**^ *P* < 0.01 (Mann–Whitney test). **b** *In situ* hybridization of miR-106b in negative control and normal cervical specimens and cervical carcinoma tissue. **c** Quantification of  *In situ* hybridization staining of 19 normal cervical specimens and 19 cervical carcinoma tissues

We evaluated the expression of miR-106b in 19 cervical cancer and 19 normal cervical samples by quantitative real-time PCR. The characteristics of patients are in Table 1. The expression of miR-106b was significantly upregulated in cervical cancer tissues, with relative mean expression 6.21(*P* < 0.01) (Fig. 1a). *In situ* hybridization revealed positive expression of miR-106b in cervical cancer tissue and interstitial tissue as compared with controls (Fig. 1b, c).

**Table 1:** Clinicopathological Characteristics of Cervical Carcinoma Patients

Factors	Case Number (*n* = 19)	(%)
Age(year)		
<=40	8	42.1
>40	11	57.9
Clinical stage		
I	9	47.4
II, III	10	52.6
Differentiation		
High	1	5.3
Moderate	3	68.4
Low	15	26.3
Pathological type		
SCC	15	78.9
AC	4	21.1
LN metastasis		
No	17	89.5
Yes	2	10.5

*SCC* Squamous Cell Carcinoma, *AC* Adenocarcinoma, *LN* Lymph nodes

### 2. miR-106b promoted the migration of cervical cancer cells

**Fig. 2:**
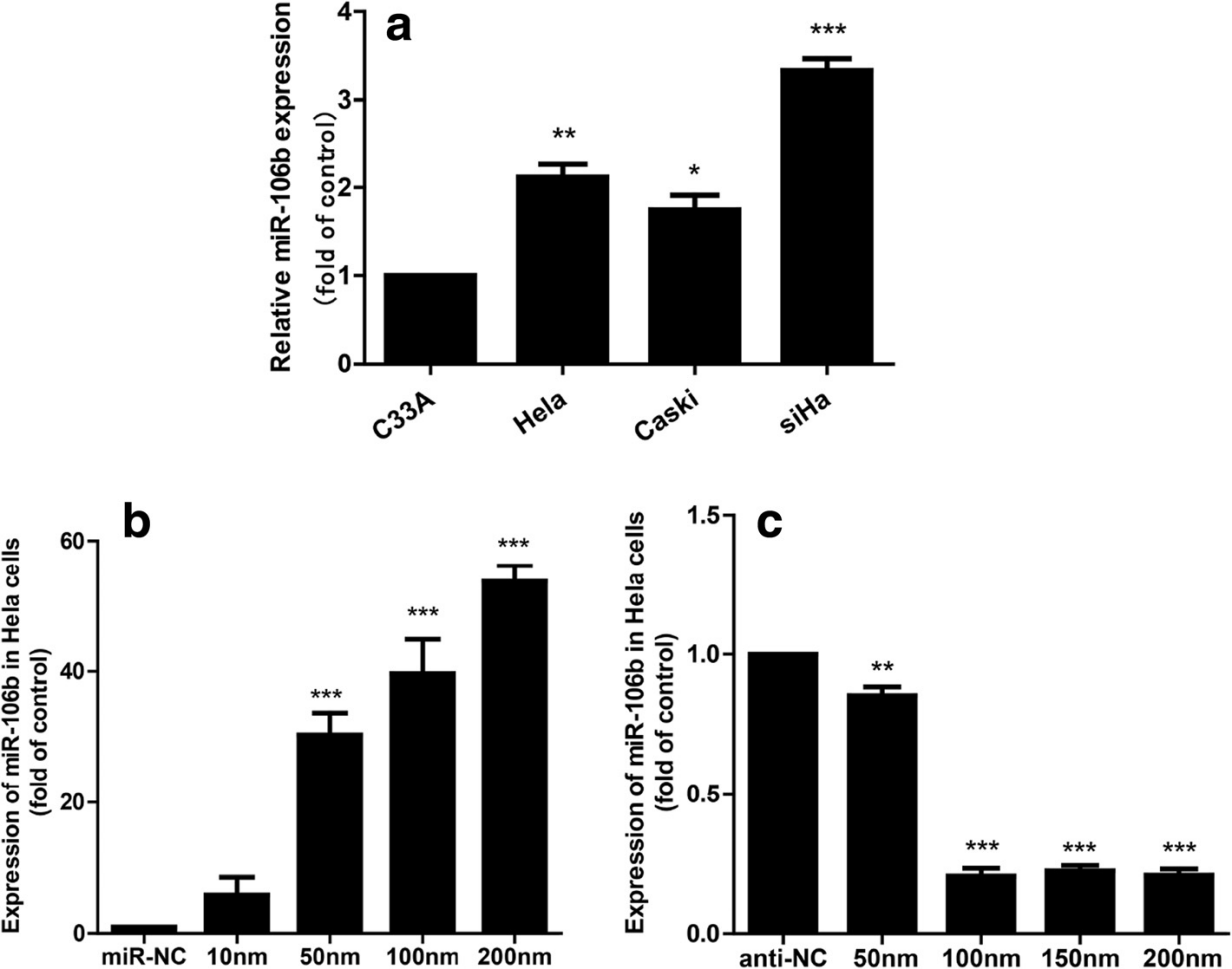
Overexpression or knockdown of miR-106b with mimic or inhibitor in cervical cancer cells. **a** Real-time PCR of the expression of miR-106b in 4 human cervical cancer cell lines. The expression of miR-106b in C33A cells was a control. *P < 0.05, **P < 0.01, *** *P* < 0.001 compared with vehicle. Data are mean ± SEM (*n* = 3). **b** miR-106b mimic (miR-106b) at four concentrations (10, 50, 100, 200 nm) was transfected into HeLa cells to overexpress miR-106b. miR-NC was a negative control.**P < 0.01, ***P < 0.001 compared with vehicle. Data are mean ± SEM (*n* = 3). **c** miR-106b inhibitor (anti-miR-106b) at four concentrations (50, 100, 150, 200 nm) was transfected into HeLa cells to knockdown miR-106b. anti- NC was a negative control.**P < 0.01, ***P < 0.001compared with vehicle. Data are mean ± SEM (*n* = 3)

miR-106b was highly expressed in the cervical cancer cell lines HeLa, CaSki and SiHa(Fig. 2a). We subsequently established effective cell models, to explore gain-of-function and loss-of-function of miR-106b in human cervical cancer. The most appropriate concentration was obtained by using different concentrations of reagent to overexpress or knockdown miR-106b with mimics or inhibitors, respectively, in HeLa cells. The miR-106b mimic (50 nm) with increasing 30 times (Fig. 2b) or miR-106b inhibitor (100 nm) with knockdown efficiency at 90 % (Fig. 2c) was the optimal concentration to over-expression or knockdown of miR-106b.

**Fig. 3:**
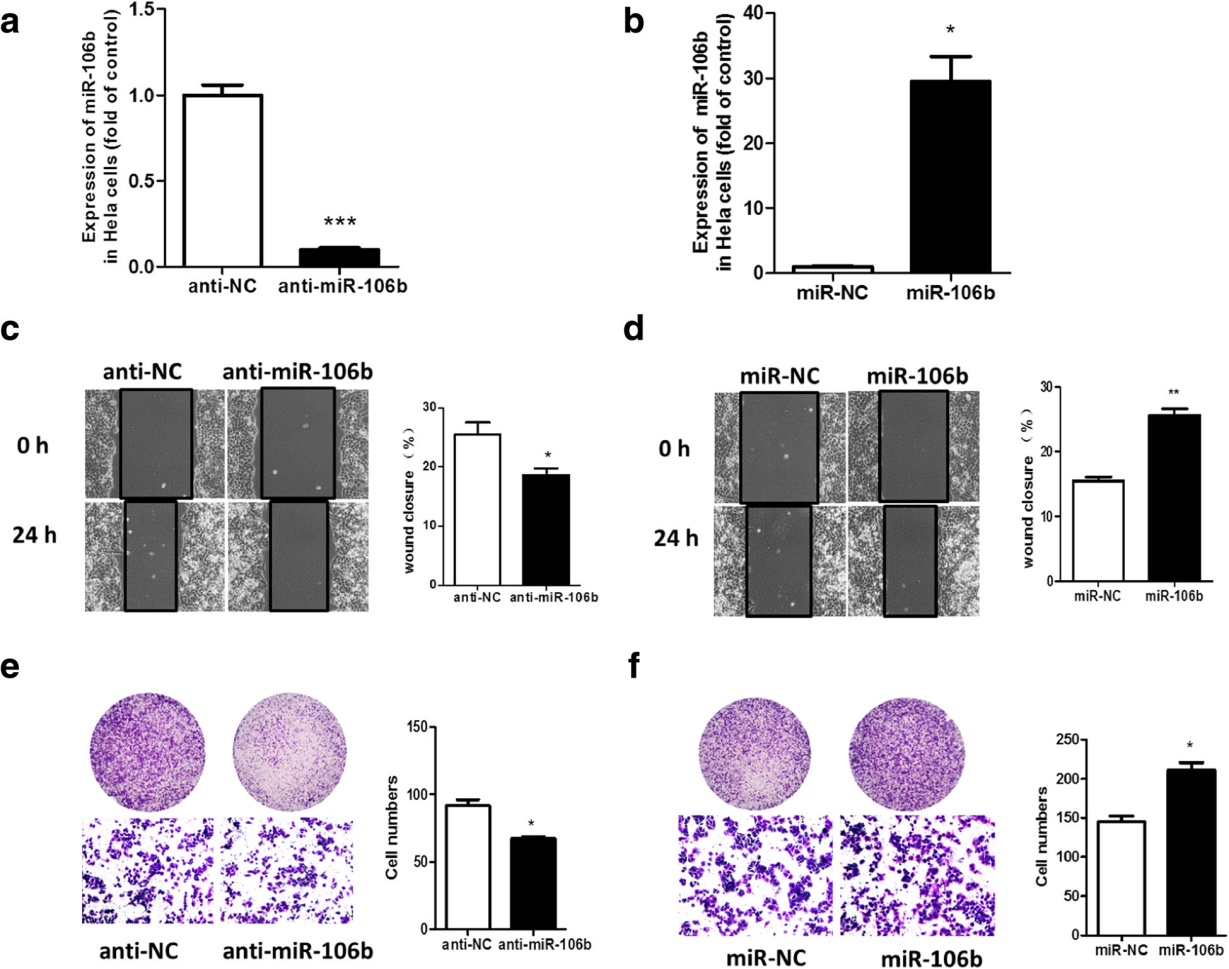
miR-106b promoted the migration of HeLa cells tested by scratch and transwell assay. HeLa cells were transfected with **a** miR-106b inhibitor (anti-miR-106b) at 100 nm and **b** miR-106b mimic(miR-106b) at 50 nm. Scratch assay of cell migration with **c** miR-106b knockdown and **d** miR-106b overexpression. Scratched cells were observed and photographed at 0 and 24 h under a microscope. Data are mean ± SEM(*n* = 3).*P < 0.05,**P < 0.01 compared to control. Transwell migration assay with **e** miR-106b knockdown and **f** miR-106b overexpression. Cells were transfected with miR-106b inhibitor(anti-miR-106b) and miR-106b control inhibitor (anti-NC). The cells on the transwell chambers were fixed, dyed, observed and photographed after culture for 24 h. Data are mean ± SEM(*n* = 3). *P < 0.05 compared to control

We overexpressed or knocked down miR-106b by transfecting the mimic 106b or inhibitor 106b, respectively, in HeLa cells and examined wound healing (Fig. 3a, b). Wound healing area was significantly decreased with miR-106b knockdown for 24 h, by 27.8 % (18.6 ± 1.16 vs 25.5 ± 1.99 cm2, *P* < 0.05) (Fig. 3c) and was enhanced with miR-106b overexpression, by 1.66-fold (25.8 ± 1.02 vs 15.5 ± 0.59 cm2, *P* < 0.01) (Fig. 3d).Transwell assay showed that cell migration was significantly reduced with miR-106b knockdown, by 26.5 % (67 ± 1 % vs 92 ± 4 %, *P* < 0.05) (Fig. 3e) and was elevated with miR-106b overexpression, by 1.46-fold (212 ± 9 vs 145 ± 7, *P* < 0.05) (Fig. 3f).

**Fig. 4:**
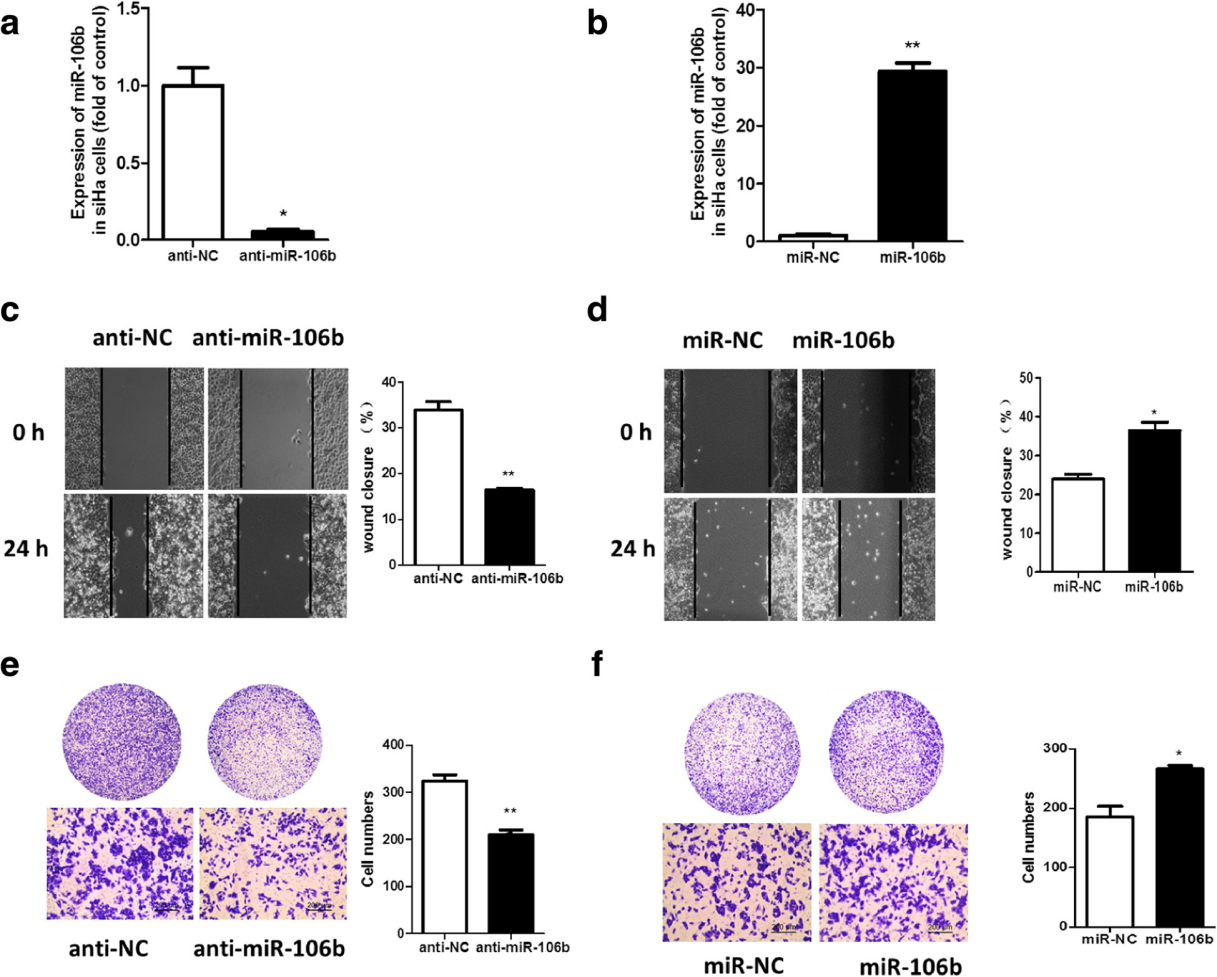
miR-106b promoted the migration of SiHa cells tested by scratch and transwell assay. SiHa cells were transfected with **a** miR-106b inhibitor (anti-miR-106b) at 100 nm. and **b** miR-106b mimics (miR-106b) at 50 nm. Scratch assay of cell migration with **c** miR-106b knockdown and **d** miR-106b overexpression. Transwell migration assay with **e** miR-106b knockdown and **f** miR-106b overexpression. Data are mean ± SEM (*n* = 3).*P < 0.05, **P < 0.01 compared to control

The role of miR-106b in SiHa cells was similar to that in Hela cells. Overexpression of miR-106b promoted SiHa cell migration, and knockdown of miR-106b inhibited the cell migration (Fig. 4).

### 3. Knockdown of miR-106b inhibited TGF-β1-induced cell migration in cervical carcinoma

**Fig. 5:**
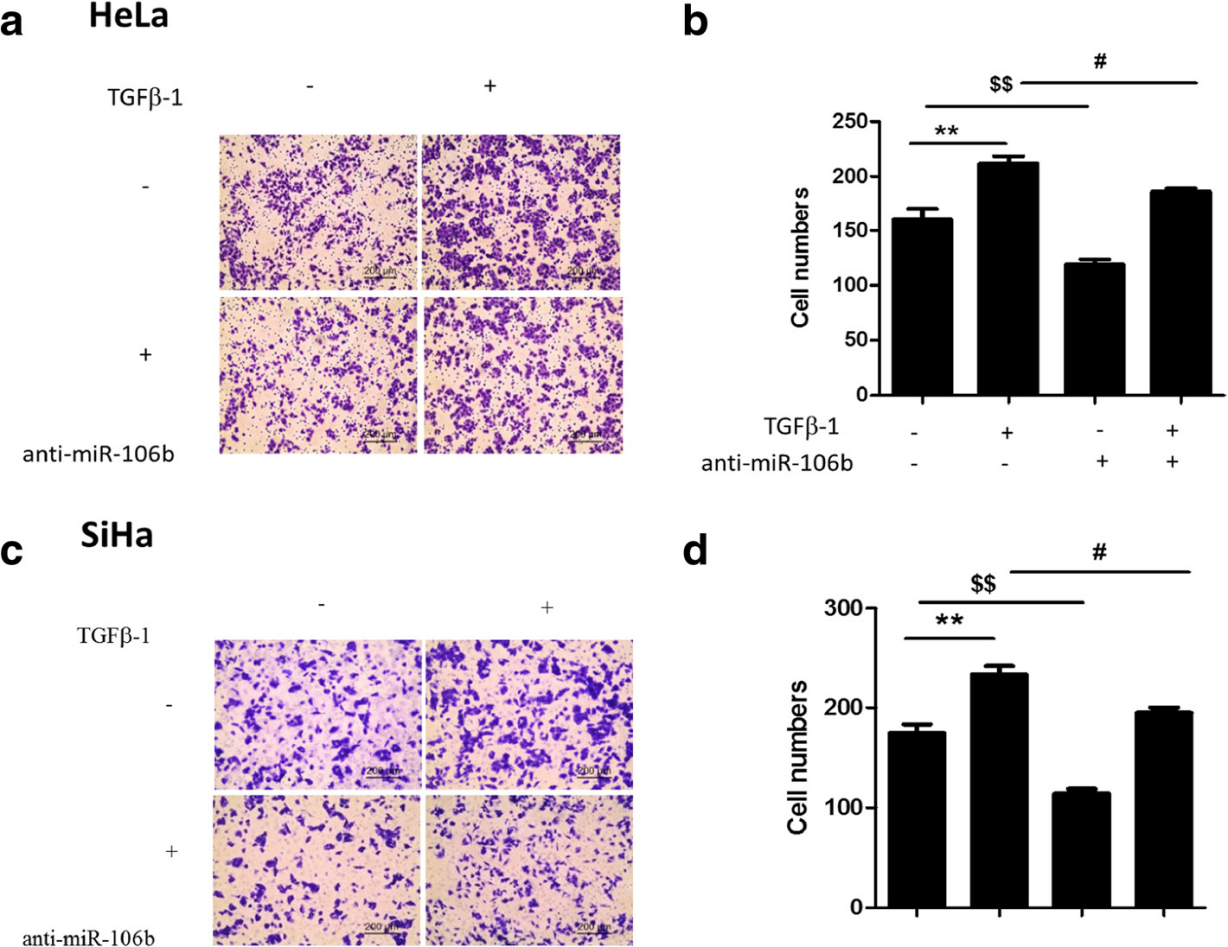
Knockdown of miR-106b inhibited TGF-β1-induced cell migration in cervical carcinoma. **a** HeLa cells were transfected with 100-nm miR-106b inhibitor (anti-miR-106b) or miR-106b control inhibitor (anti-NC) for 24 h, then migration was induced with or without TGF-β1 (5 ng/mL) for 24 h. **b** Quantification of A.**P < 0.01, ^$$^ *P* < 0.01, ^#^ *P* < 0.05. **c** SiHa cells were transfected with 100-nm miR-106b inhibitor (anti-miR-106b) or miR-106b control inhibitor (anti-NC) for 24 h, then migration was induced with or without TGF-β1 (5 ng/mL) for 24 h. **d** Quantification of C.**P < 0.01, ^$$^ *P* < 0.01, ^#^ *P* < 0.05

TGF-β1 plays an important role in cervical cancer progression. Whether miR-106b is involved in TGF-β1-induced migration of cervical cancer cells is unknown. Our data showed that HeLa and SiHa cell migration was increased with TGF-β1 as compared with controls (Fig. 5) (*P* < 0.01). After inhibition of miR-106b, the number of cells migration was greatly reduced as compared with cells, treated with TGF-β1 alone (Fig. 5) (*P* < 0.05).

### 4. DAB2 was regulated by TGF-β1 and miR-106b

To understand how miR-106b was involved in TGF-β1-induced cell migration in cervical carcinoma, we identified the target genes of miR-106b by using TargetScan. The TargetScan predictive algorithm identified DAB2 as a potential target of miR-106b.

DAB2 has low expression in many human cancers and was regulated by TGF-β1 in previous studies. Furthermore, the relationship between DAB2 and miR-106b remains unknown.

**Fig. 6:**
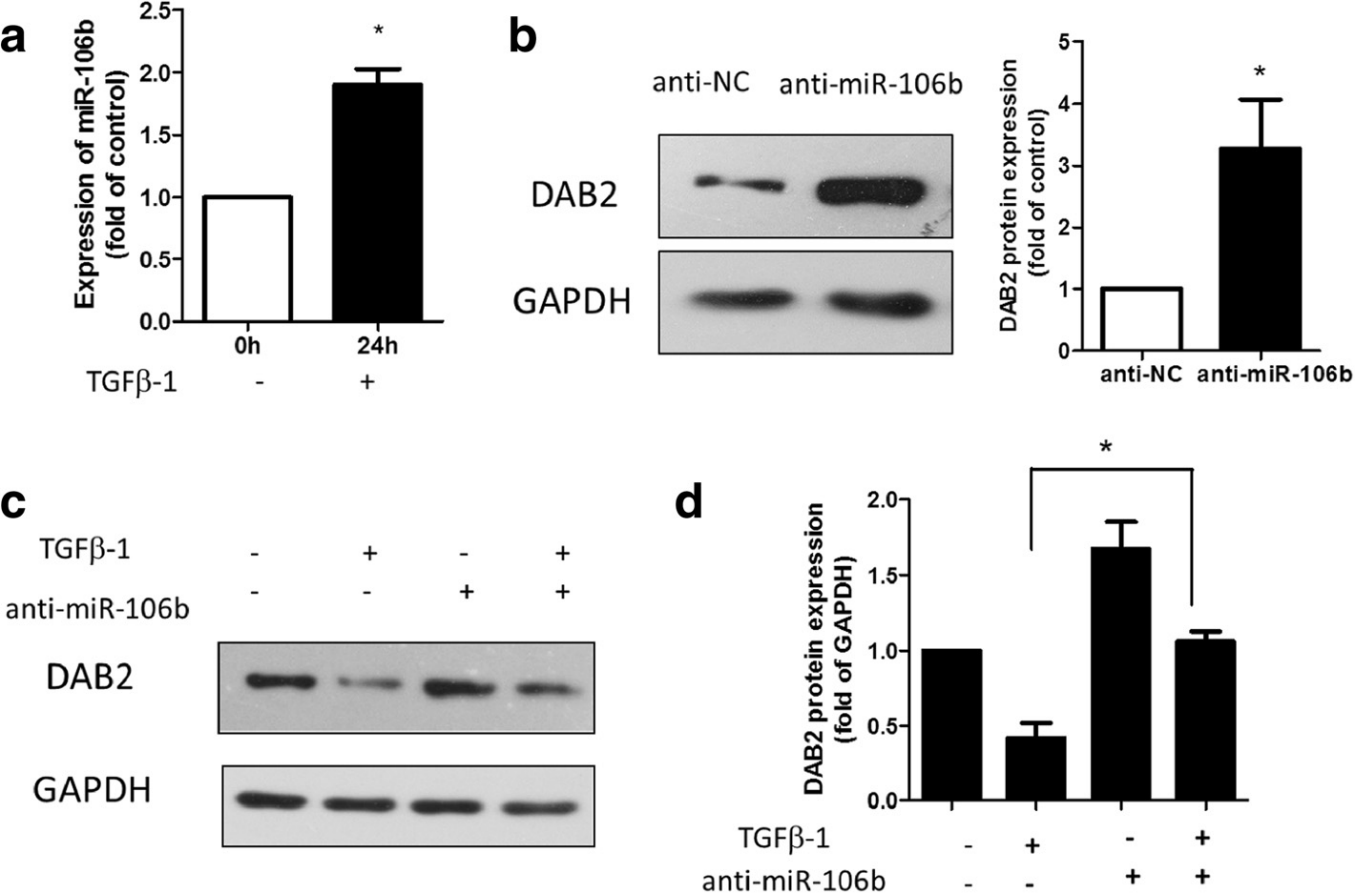
DAB2, a predicted target gene of miR-106b, was inhibited by TGF-β1 in part via miR-106b. **a** Real-time PCR analysis of mRNA expression of miR-106b in HeLa cells with TGF-β1 treatment for 24 h. Data are mean ± SEM (*n* = 3). ^*^ *P* < 0.01 compared to control. **b** Western blot analysis of protein level of DAB2 in HeLa cells transfected with miR-106b inhibitor (anti-miR-106b) or miR-106b control inhibitor (anti-NC). Data are mean ± SEM (*n* = 3). *P < 0.05 compared to control. **c** Western blot analysis of DAB2 protein level in HeLa cells transfected with 100 nm miR-106b inhibitor (anti-miR-106b) for 24 h, then with or without TGF-β1 (5 ng/mL) for 24 h; GAPDH was an internal control. **d** Quantification of C.*P < 0.05

We next identified the regulation of TGF-β1, miR-106b and DAB2. The expression of miR-106b was doubled with TGF-β1 treatment for 24 h (Fig. 6a). As well, the expression of DAB2 protein was increased with knockdown of miR-106b in HeLa cells, for an inverse relation (Fig. 6b). We further detected DAB2 expression with TGF-β1 treatment and miR-106b inhibition. DAB2 protein expression was decreased with TGF-β1 treatment. The TGF-β1 downregulated expression of DAB2 protein was closely related to miR-106b expression (Fig. 6c, d).

### 5. DAB2 was involved in TGF-β1-induced cell migration in cervical carcinoma

**Fig. 7:**
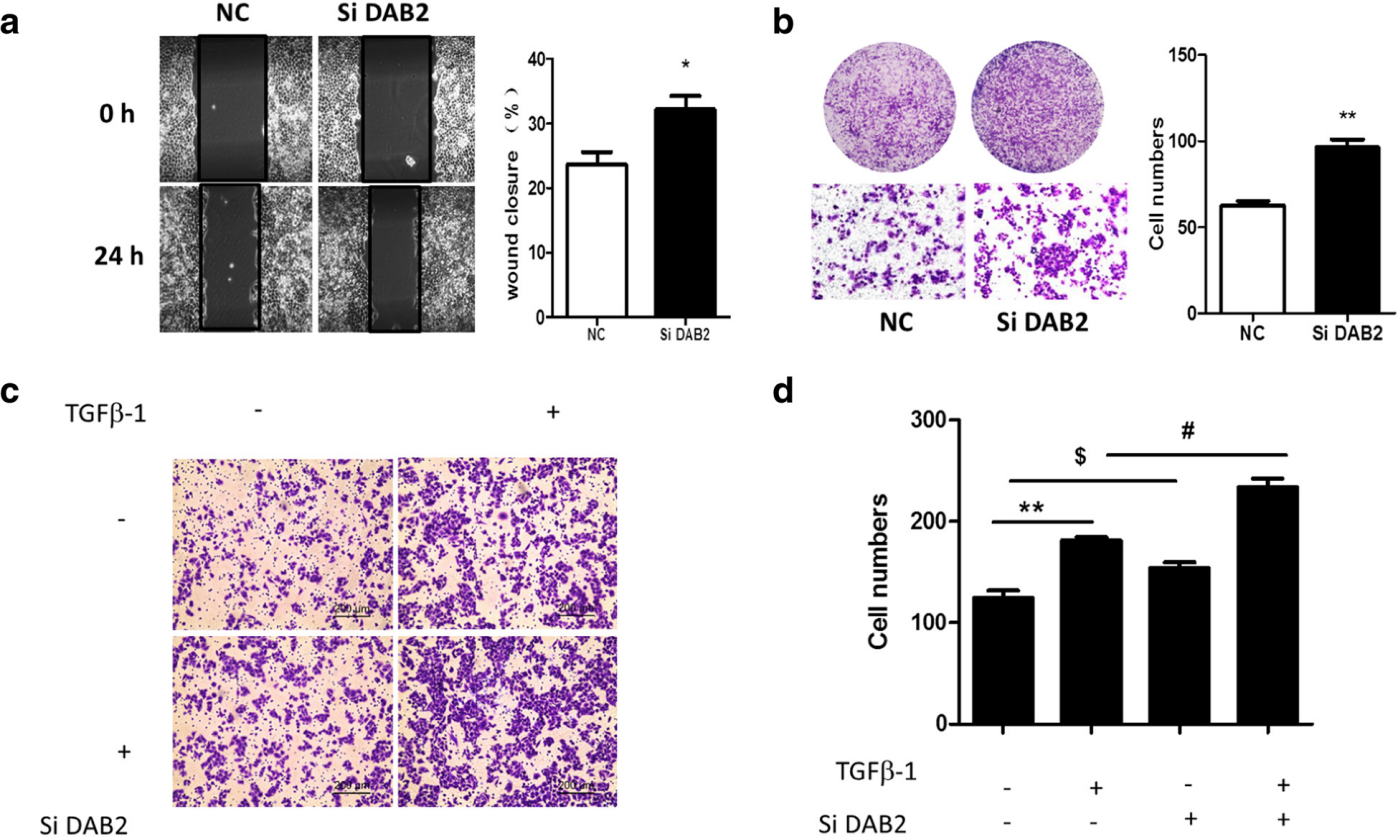
Knockdown of DAB2 promoted TGF-β1-induced cell migration in cervical carcinoma. **a** Scratch assay of cell migration in cells transfected with DAB2-NC or DAB2 siRNA at 0 and 24 h. Data are mean ± SEM (*n* = 3).*P < 0.05 compared to control. **b** Transwell migration assay of cells transfected with DAB2-NC or DAB2 siRNA at 24 h. Data are mean ± SEM(*n* = 3).^*^ *P* < 0.01 compared to control. **c** HeLa cells were transfected with DAB2-NC or DAB2 siRNA for 24 h, then migration was induced with or without TGF-β1 (5 ng/mL) for 24 h and quantification. **d** Quantification of C. **P < 0.01, ^$^ *P* < 0.05, ^#^ *P* < 0.05

We further explored the role of DAB2 in TGF-β1-induced cell migration. The ability for cell migration was increased with siRNA knockdown of DAB2, compared with controls (Fig. 7a, b) (*P* < 0.01). After treatment with TGF-β1, the number of migrating cells was obviously elevated as compared with TGF-β1 alone (Fig. 7c, d) (*P* < 0.05).

### 6. Identification of DAB2 as a miR-106b-directed target gene

**Fig. 8:**
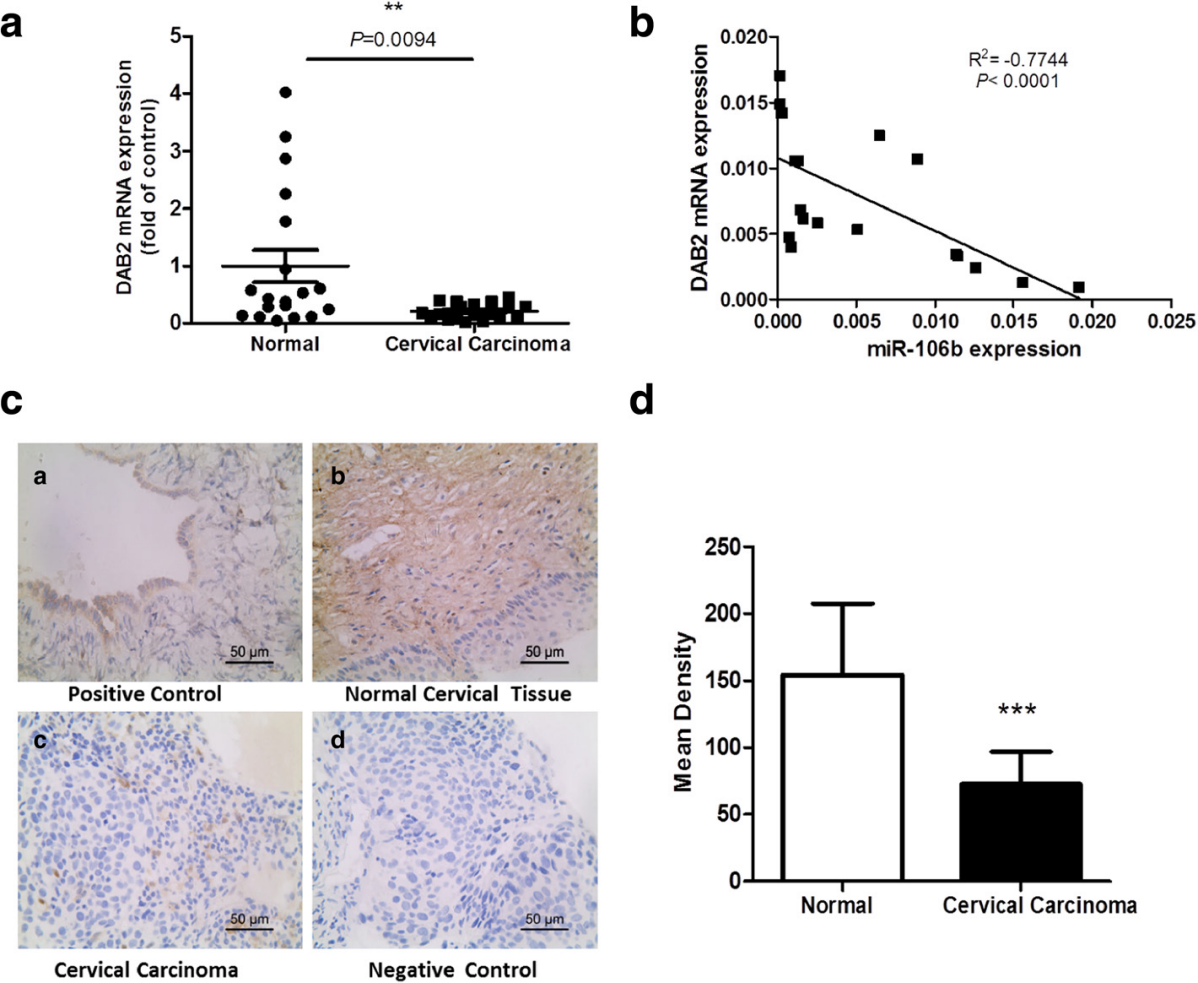
DAB2 had low expression in cervical cancer tissues, and which was negatively correlated with miR-106b expression. **a** The expression of DAB2 in 19 human cervical carcinoma tissues and 19 normal cervical tissues (**P < 0.01) (nonparametric test with Mann–Whitney test). U6 small nuclear RNA was an internal control. Each point represents 1 sample. The horizontal bar is the mean and whiskers are SEM. **b** Spearman correlation analysis of DAB2 and miR-106b expression in 19 cervical cancer tissues.***P < 0.0001, R2 = −0.7744. **c** Immunohistochemistry of protein expression of DAB2 in cervical tissues. Expression of DAB2 protein in a) normal ovarian tissue as a positive control, b) normal cervical tissues, c) cervical carcinoma tissues, and d) negative control. **d** Quantification of immunohistochemical staining of 19 normal cervical specimens and 19 cervical carcinoma tissues.**P < 0.01

We tested the expression of DAB2 in cervical tissues. The mRNA expression of DAB2 in 19 cervical carcinoma samples was significantly reduced, by 79.1 %, to 0.21 ± 0.03, as compared with normal cervical tissues (*P* < 0.01) (Fig. 8a). miR-106b level was negatively correlated with DAB2 level (R^2^ = −0.7744, *P* < 0.001) (Fig. 8b). Immunohistochemistry revealed reduced DAB2 protein level in cervical cancer tissue (Fig. 8c, d).

**Fig. 9:**
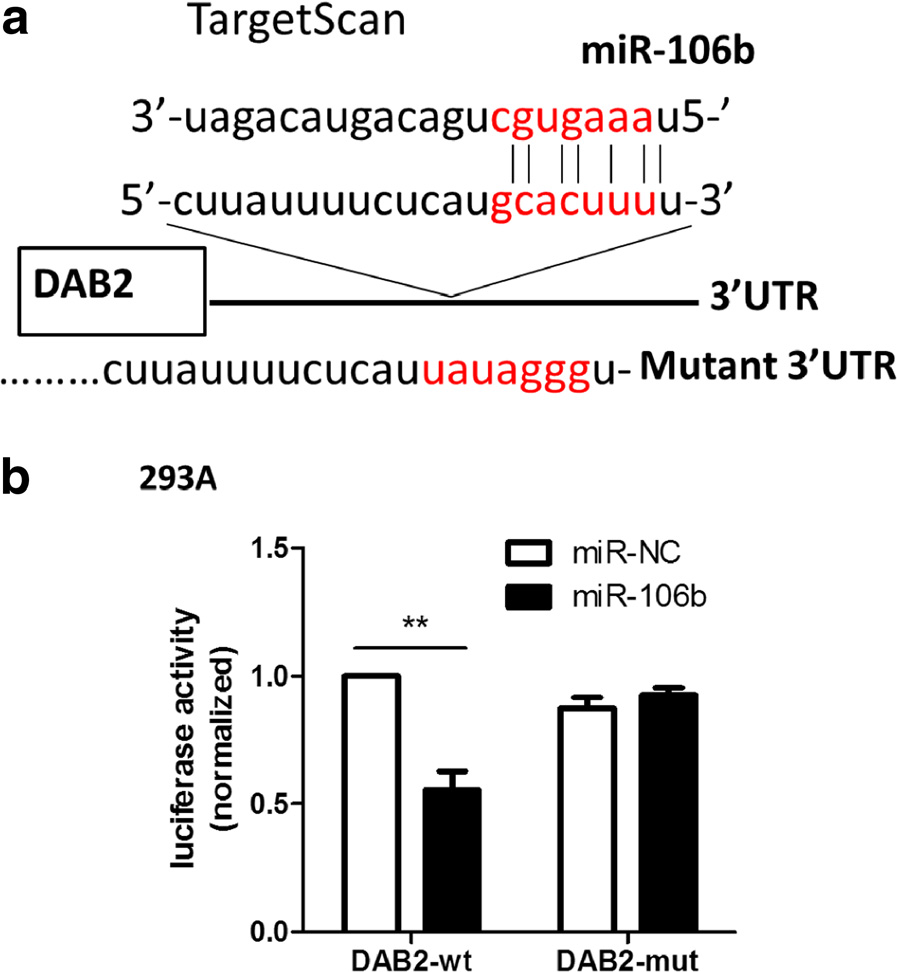
DAB2 was identified as a miR-106b-directed target gene. **a** The predicted interaction site of miR-106b and candidate target gene DAB2 3′UTR. **b** Luciferase assay of HEK-293A cells co-transfected with miR-106b mimic and pGL-3-DAB2 plasmid (miR-NC and miR-106b with DAB2-wt 3′UTR; miR-NC and miR-106b with DAB2-mut 3′UTR) after 24 h. Data are mean ± SEM(*n* = 3).**P < 0.01

To identify whether DAB2 is a miR-106b–directed target gene, we predicted the interaction site of 7 bp of miR-106b and the candidate target gene DAB2 3′UTR (Fig. 9a). The wild-type (WT) or mutant (Mut) DAB2 3′UTR was inserted into the region downstream of the Renilla luciferase gene in the pmir-GLO vector. On co-transfection with miR-106b and DAB2-WT 3′UTR in HEK-293A cells, luciferase activity was lower, by 55.4 %, to 0.55 ± 0.07, as compared with controls (*P* < 0.01). However, on co-transfection with Mut-DAB2-3′UTR, luciferase activity did not differ from the control (Fig. 9b).

### 7. Working model

**Fig. 10:**
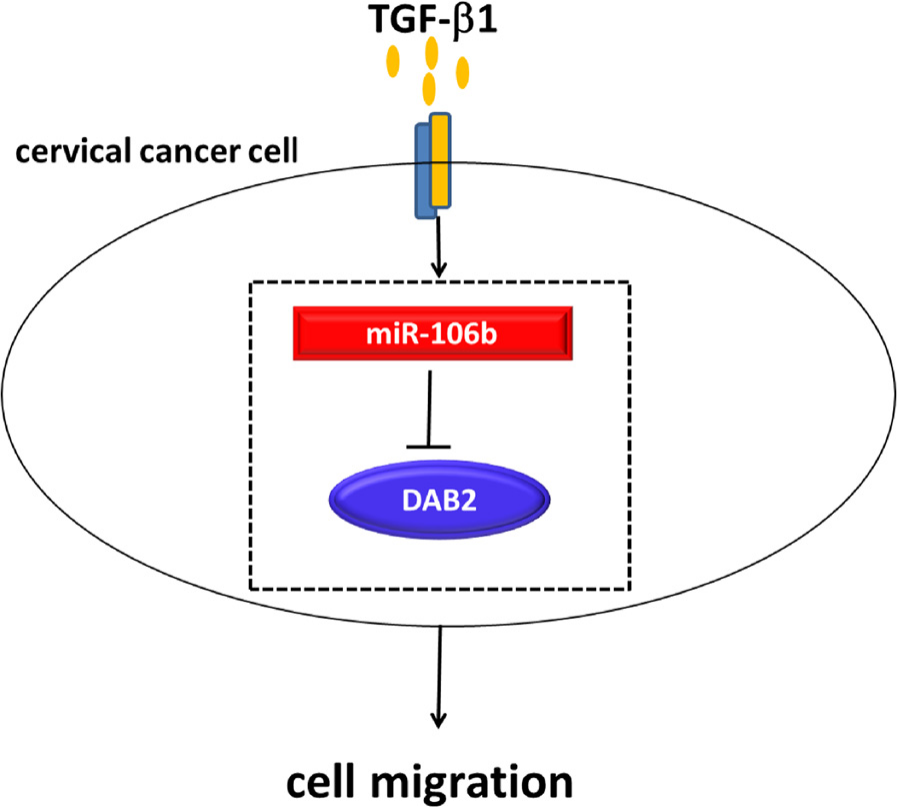
miR-106b is involved in TGF-β1-induced cell migration by targeting DAB2 in cervical carcinoma. The Large circle represents one cervical cancer cell. The orange small circles represent many TGF-β1 cell factors. The two small rectangular blocks on the circle indicate the receptors of TGF-β1. The red rectangle represents miR-106b. The blue oval represents DAB2 protein

The working model of miR-106b in cervical cancer is shown in Fig. 10. miR-106b can directly target the degradation of DAB2 protein. TGF-β1 inhibits the expression of DAB2 protein by up-regulating miR-106 partly. The TGF-β1/miR-106b/DAB2 axis is involved in the migration of cervical cancer cells. In general, miR-106b is involved in TGF-β1-induced cell migration by targeting DAB2 in cervical carcinoma.

## Discussion

The main factors affecting the clinical prognosis of metastatic and recurrent cervical cancer have not been revealed completely. Understanding the molecular mechanism related to cancer processes is crucial to find better strategies for refractory cervical cancer treatment. miRNAs have been found to play a vital role in tumor progression [6–9]. miR-106b was identified to be a tumor promoting factor in breast cancer, hepatocellular carcinoma and melanoma [16–18]. In gastric cancer, miR-106b activates PI3K/AKT signaling by inhibiting the expression of the tumor suppressor gene PTEN, which leads to the down-regulation of cell adhesion molecule E-cadherin, thereby promoting the migration of cancer cells [11, 19]. However, the role of miR-106b in cervical cancer has not been investigated. Our previous study first found that miR-106b was a key factor in cervical cancer development [10]. This study showed that miR-106b was significantly upregulated in cervical cancer tissues as compared with control tissue, and miR-106b overexpression promoted the migration of HeLa and SiHa cells, which suggests that miR-106b may have a critical role in regulating infiltration and metastasis of cervical cancer. Our data confirmed that miR-106b acts as a tumor promoter in cervical cancer progression.

Accumulating evidence has indicated that multiple factors are involved in the invasion and metastasis of cervical cancer, including human papilloma virus protein, soluble cytokine and its ligand, cytoskeletal regulatory factors, integrin and matrix metalloproteinase [20]. Among them, TGF-β1 is an important regulatory factor in cervical cancer. TGF-β1 has been reported to be involved in the EMT, focal adhesion kinase (FAK) activation, integrin αvβ3-induced expression at the cell surface, enhanced adhesion between cells and the surrounding tissue, and increased migration and invasion in cervical cancer cells [21]. We found out whether miR-106b was also involved in the progress of TGF-β1-induced migration in cervical cancer cells. miR-106b inhibitor treatment decreased the TGF-β1-stimulated migration of cervical cancer cells. Previous study found that miR-106b enhanced the migration in breast cancer cells by activating the TGF-β/Smad signaling pathway via degradation of Smad7 [13]. Our findings are consistent with conclusions for breast cancer. Moreover, we revealed the molecular mechanisms of miR-106b involved in TGF-β1-induced cervical cancer cell migration.

miRNAs negatively regulate their target mRNAs by the RISC complex, which leads to mRNA translation inhibition or degradation, for a wide variety of functions.

We then found target genes of miR-106b according to the network map from our miRNA and mRNA microarray assays. In these predicted target genes, DAB2 is considered a candidate tumor suppressor gene for its absent and reduced expression in various tumors [22–25]. Moreover, downregulation of DAB2 could enable TGF-β-mediated cell motility and tumor growth in vivo [23, 26]. In this study, DAB2 was first identified to have low expression in cervical cancer. As well, DAB2 protein level was downregulated with TGF-β1 treatment for 24 h. miR-106b was confirmed to be involved in the effect of TGF-β1 by depleting DAB2 protein. To be excited, knockdown of DAB2 enhanced TGF-β1-induced migration in HeLa cells.

Another important finding from this study was that miR-106b directly targeted the 3′UTR of the DAB2 and inhibited DAB2 expression as seen on double luciferase reporter gene assay. As well, DAB2 expression was negatively correlated with miR-106b expression in clinical tissue samples and cell experiments. Hannigan et al. found downregulation of DAB2 mRNA expression in human squamous cell carcinoma and that DAB2 level was an independent predictor of metastasis and poor prognosis [26]. Studies showed that DAB2 protein as a tumor suppressor inhibits cell growth in lung cancer, and miR-93, which is homologous to miR-106b, can inhibit its function in regulating the DAB2 protein level [27]. These studies support our findings in different aspects.

## Conclusions

miR-106b is regulated by TGF-β1 and contributes to cell migration by targeting DAB2 in cervical carcinoma. The TGF-β1/miR-106b/DAB2 axis could provide further insight into the pathogenesis of cervical carcinoma, and miR-106b could be a biomarker and potential therapeutic target in cervical cancer.

## Abbreviations

miRNAs: microRNAs
TGF-β: transforming growth factor-β
DAB2: disabled homolog 2
PTEN: phosphatase and tensin homolog
WT: wild-type
Mut: mutant
MCM7: minichromosome maintenance complex component 7
SCC: squamous cell carcinoma
AC: adenocarcinoma
LN: lymphnode

## References

[1] Arbyn M, Castellsagué X, de Sanjosé S, Bruni L, Saraiya M, Bray F (2011). Worldwide burden of cervical cancer in 2008. Ann Oncol.

[2] Jiang L, Zeng Y, Li J, Wang H, Xia Y, Fang X (2011). Performance of high-risk human papillomavirus testing in the triage of abnormal cervical cytology among Chinese younger women in Shanghai, China. Asian Pac J Cancer Prev.

[3] Chen W, Zheng R, Zeng H, Zhang S, He J (2015). Annual report on status of cancer in China, 2011. Chin J Cancer Res.

[4] Wright JD, Grigsby PW, Brooks R, Powell MA, Gibb RK, Gao F (2007). Utility of parametrectomy for early stage cervical cancer treated with radical hysterectomy. Cancer.

[5] Yuan SH, Liang XF, Jia WH, Huang JL, Wei M, Deng L (2008). Molecular diagnosis of sentinel lymph node metastases in cervical cancer using squamous cell carcinoma antigen. Clin Cancer Res.

[6] Tang F, Kaneda M, O’Carroll D, Hajkova P, Barton SC, Sun YA (2007). Maternal microRNAs are essential for mouse zygotic development. Genes Dev.

[7] Tay YM, Tam WL, Ang YS, Gaughwin PM, Yang H, Wang W (2008). MicroRNA-134 modulates the differentiation of mouse embryonic stem cells, where it causes post-transcriptional attenuation of Nanog and LRH1. Stem Cells.

[8] Chan SH, Wu CW, Li AF, Chi CW, Lin WC (2008). miR-21 microRNA expression in human gastric carcinomas and its clinical association. Anticancer Res.

[9] Kogo R, How C, Chaudary N, Bruce J, Shi W, Hill RP (2015). The microRNA-218 ~ Survivin axis regulates migration, invasion, and lymph node metastasis in cervical cancer. Oncotarget.

[10] Ma D, Zhang YY, Guo YL, Li ZJ, Geng L (2012). Profiling of microRNA-mRNA reveals roles of microRNAs in cervical cancer. Chin Med J.

[11] Yang TS, Yang XH, Chen X, Wang XD, Hua J, Zhou DL (2014). MicroRNA-106b in cancer-associated fibroblasts from gastric cancer promotes cell migration and invasion by targeting PTEN. FEBS Lett.

[12] Gong C, Qu S, Lv XB, Liu B, Tan W, Nie Y (2014). BRMS1L suppresses breast cancer metastasis by inducing epigenetic silence of FZD10. Nat Commun.

[13] Smith AL, Iwanaga R, Drasin DJ, Micalizzi DS, Vartuli RL, Tan AC (2012). The miR-106b-25 cluster targets Smad7, activates TGF-β signaling, and induces EMT and tumor initiating cell characteristics downstream of Six1 in human breast cancer. Oncogene.

[14] Fullár A, Dudás J, Oláh L, Hollósi P, Papp Z, Sobel G (2015). Remodeling of extracellular matrix by normal and tumor-associated fibroblasts promotes cervical cancer progression. BMC Cancer.

[15] Nagura M, Matsumura N, Baba T, Murakami R, Kharma B, Hamanishi J (2015). Invasion of uterine cervical squamous cell carcinoma cells is facilitated by locoregional interaction with cancer-associated fibroblasts via activating transforming growth factor-beta. Gynecol Oncol.

[16] Gong C, Qu S, Liu B, Pan S, Jiao Y, Nie Y (2015). MiR-106b expression determines the proliferation paradox of TGF-beta in breast cancer cells. Oncogene.

[17] Li BK, Huang PZ, Qiu JL, Liao YD, Hong J, Yuan YF (2014). Upregulation of microRNA-106b is associated with poor prognosis in hepatocellular carcinoma. Diagn Pathol.

[18] Prasad R, Katiyar SK (2014). Down-regulation of miRNA-106b inhibits growth of melanomacells by promoting G1-phase cell cycle arrest and reactivationof p21/WAF1/Cip1 protein. Oncotarget.

[19] Stewart AL, Mhashilkar AM, Yang XH, Ekmekcioglu S, Saito Y, Sieger K (2002). PI3 kinase blockade by Ad-PTEN inhibits invasion and induces apoptosis in RGP and metastatic melanoma cells. Mol Med.

[20] Hellner K, Mar J, Fang F, Quackenbush J, Münger K (2009). HPV16 E7 oncogene expression in normal human epithelial cells causes molecular changes indicative of an epithelial to mesenchymal transition. Virology.

[21] Yi JY, Hur KC, Lee E, Jin YJ, Arteaga CL, Son YS (2002). TGFbeta1 -mediated epithelial to mesenchymal transition is accompanied by invasion in the SiHa cell line. Eur J Cell Biol.

[22] Mok SC, Chan WY, Wong KK, Cheung KK, Lau CC, Ng SW (1998). DOC-2, a candidate tumor suppressor gene in human epithelial ovarian cancer. Oncogene.

[23] Martin JC, Herbert BS, Hocevar BA (2010). Disabled-2 downregulation promotes epithelial-to-mesenchymal transition. Br J Cancer.

[24] Fulop V, Colitti CV, Genest D, Berkowitz RS, Yiu GK, Ng SW (1998). DOC-2/hDab2, a candidate tumor suppressor gene involved in the development of gestational trophoblastic diseases. Oncogene.

[25] Zhou J, Scholes J, Hsieh JT (2003). Characterization of a novel negative regulator (DOC-2/DAB2) of c-Src in normal prostatic epithelium and cancer. J BiolChem.

[26] Hannigan A, Smith P, Kalna G, Lo Nigro C, Orange C, O’Brien DI (2010). Epigenetic downregulation of human disabled homolog 2 switches TGF-beta from a tumor suppressor to a tumor promoter. J Clin Invest.

[27] Du L, Zhao Z, Ma X, Hsiao TH, Chen Y, Young E (2014). miR-93-directed downregulation of DAB2 defines a noveloncogenic pathway in lung cancer. Oncogene.

